# Associations of circulating insulin-like growth factor-1 and insulin-like growth factor binding protein-3 with the expression of stem cell markers in benign breast tissue

**DOI:** 10.1186/s13058-025-02002-z

**Published:** 2025-04-07

**Authors:** Lusine Yaghjyan, Yujing J. Heng, Brian R. Sardella, Divya Murthy, Matt B. Mahoney, Bernard Rosner, Kornelia Polyak, Maisey Ratcliff, Rulla M. Tamimi

**Affiliations:** 1 Department of Epidemiology, College of Public Health and Health Professions and College of Medicine, University of Florida, 2004 Mowry Rd., Gainesville, FL 32610 USA; 2 Department of Pathology, Beth Israel Deaconess Medical Center, Harvard Medical School, Boston, MA USA; 3 Channing Division of Network Medicine, Department of Medicine, Brigham and Women’s Hospital and Harvard Medical School, Boston, MA USA; 4 Dana-Farber Cancer Institute, Brigham and Women’s Hospital, and Harvard Medical School, Boston, MA USA; 5 Department of Population Health Sciences, Weill Cornell Medicine, New York, NY USA

**Keywords:** Benign breast disease, Breast cancer risk, Stem cell markers

## Abstract

**Background:**

The insulin-like growth factor (IGF) pathway is implicated in a naturally occurring process of tissue remodeling during which cells acquire stem cell-like characteristics. We examined associations of circulating IGF-1 and IGF binding protein-3 (IGFBP-3) with expression of CD44, CD24, and ALDH1A1 stem cell markers in benign breast biopsies.

**Methods:**

This study included 151 cancer-free women with incident biopsy-confirmed benign breast disease and blood samples within the Nurses’ Health Study II. The data on reproductive and other BCa risk factors were obtained from biennial questionnaires. Immunohistochemistry (IHC) was done on tissue microarrays. For each core, the IHC expression was assessed using QuPath, and expressed as % of cells that stain positively for a specific marker out of the total cell count. Generalized linear regression was used to examine the associations of plasma IGF-I and IGFBP-3 (continuous log-transformed and quartiles) with log-transformed expression of each marker (in epithelium and stroma), adjusted for BCa risk factors.

**Results:**

In multivariate analysis, continuous circulating IGF-1 and IGFBP-3 measures were not associated with the continuous expression of any of the markers in the epithelium or stroma. Women whose IGFBP-3 levels were in the top quartile appeared to have lower expression of stromal CD24 compared to those in the lowest quartile (β = − 0.38, 95% CI − 0.69, − 0.08, *p*-trend = 0.06).

**Conclusions:**

Higher circulating IGFBP-3 levels were associated with lower stromal CD24 expression in benign breast tissue. Our findings provide indirect evidence of the inducing effect of IGF pathway on epithelial-to-mesenchymal transitions and stem cell activity in the breast.

**Supplementary Information:**

The online version contains supplementary material available at 10.1186/s13058-025-02002-z.

## Background

Breast stem cells play a critical role in sustaining breast tissue architecture throughout the woman’s life [1, 2]. However, potentially limitless self-renewal capacity and high susceptibility to various endogenous and exogenous mutagenic insults increase the chances of their tumorigenic transformation [1, 3]. According to the stem cell hypothesis of breast carcinogenesis, breast cancer development might be directly related to the size and mitotic activity of the stem cell pool in the breast [4]. Further, in the breast, stem cells are the only cell subpopulation that can accumulate all the oncogenic alterations [1].

Insulin-like growth factors (IGF) play an important role in the structure of breast lobules and lobular involution [5]. Epidemiologic and animal studies suggest an increase in breast cancer risk with elevated circulating IGF-1 and insulin-like growth factor-binding protein 3 (IGFBP-3) which are also associated with a number of breast cancer risk factors, including birthweight, mammographic density, benign breast disease (BBD) as well as BBD to tumor progression [5–11]. IGF-1 system plays an important role in breast carcinogenesis [9, 12] by regulating the epithelial-to-mesenchymal transition (EMT, a natural process for tissue remodeling and wound healing), and stem cell-related processes across several tissues [13, 14]. An overlap between EMT and stem cell mechanisms is supported by the evidence that cells undergoing EMT acquire stem cell-like characteristics including self-renewal, gain of specific gene expression patterns, and ability to initiate tumorigenesis [13, 15]. However, the relationship between circulating IGF pathway markers and stem cells has never been investigated. To address this knowledge gap, we examined associations of IGF-1 and IGFBP-3 with well-characterized stem cell markers CD44, CD24, and ALDH1A1. There are no universal markers for breast cancer stem cells (and normal mammary stem cells), but CD44, CD24, and ALDH1A1 stem cell markers remain the most accepted markers that have been validated and linked to younger age at diagnosis, higher odds of unfavorable tumor characteristics, and poor prognosis and chemotherapy resistant breast cancer [16–24]. Accumulating reports show that breast cancer stem cells with co-stained CD44^+^/CD24^−^ or CD44^+^/CD24^low^ and ALDH1^+^ or ALDH1^high^ are responsible for tumor initiation, progression, metastasis, and drug resistance [16–24]. In line with this evidence, we hypothesized that higher levels of IGF-1 and IGFBP-3 would be positively associated with the expression of CD44 and ALDH1A1, and inversely associated with CD24 (consistent with associations of CD44high, ALDH1A1high, and CD24low with unfavorable tumor features).

## Materials and methods

### Study population

Our analysis included women with incident biopsy-confirmed benign breast disease (BBD) in the Nurses’ Health Study II (NHSII) cohort. NHSII followed registered nurses in the United States who were 25–42 years old at enrollment. After administration of the initial 1989 NHS II questionnaire, the information on breast cancer risk factors (body mass index [BMI], reproductive history, menopausal hormone therapy [MHT] use, and alcohol use) and any diagnoses of cancer or other diseases (including BBD) was updated through biennial questionnaires which were then confirmed via medical record review [25]. The initial questionnaire and all subsequent biennial questionnaires asked participants to report any diagnosis of BBD and to indicate whether it was confirmed by biopsy or aspiration. Details of this incident BBD study and the BBD assessment have been previously described [12, 26]. We obtained BBD pathology records and archived biopsy specimens for all BBD cases from their hospital pathology departments. Women were excluded if they had evidence of in situ or invasive carcinoma or unknown lesion type at the time of benign breast biopsy.

Between 1996 and 1999, 29,611 NHSII participants who were 32–45 years old provided a blood sample [27, 28]. In brief, premenopausal NHSII participants who were not using any hormones, were not pregnant, or have not breastfed in the previous 6 months, provided a 30-ml blood sample collected 7–9 days before the anticipated start of their next menstrual cycle (luteal blood draw). NHSII women who were ineligible to provide timed samples (i.e., perimenopausal, postmenopausal, had a simple hysterectomy, currently used any hormones, or declined to give timed samples) provided a single 30-ml blood sample (referred to as “untimed” samples, 22.5% of women in our final study sample). For both luteal and untimed samples, women shipped the blood to the Brigham and Women’s/Harvard Cohorts Biorepository laboratory, with an ice-pack, via overnight courier, where the samples were processed, separated into plasma, red blood cell, and white blood cell components, and aliquoted into labeled cryotubes. All samples have been stored in the vapor phase of continuously monitored liquid nitrogen freezers (below − 130 °C) since collection.

In the current analysis, we included 151 women who had complete data on breast cancer factors, IGF measures, and staining results for stem cell markers. All women in our sample were diabetes-free at the time of the blood collection. The study protocol was approved by the institutional review boards (IRB) of the Brigham and Women’s Hospital and Harvard T.H. Chan School of Public Health, and those of participating registries as required, and University of Florida IRB. Consent was obtained or implied by return of questionnaires.

### Benign breast biopsy confirmation and BBD subtypes

Hematoxylin and eosin (H&E) breast tissue slides were retrieved for biopsy-confirmed BBD patients who gave permission to review their biopsy records. The slides were previously independently reviewed by one of three pathologists in a blinded fashion, i.e. the evaluating pathologists were blinded to type of BBD noted on the original diagnosis [29, 30]. Any slide identified as having either questionable atypia or atypia was jointly reviewed by two pathologists [29, 30]. Each benign breast biopsy was classified according to the categories of Page et al.[31] as non-proliferative, proliferative without atypia, or atypical hyperplasia (ductal or lobular hyperplasia) [12].

### Tissue microarray (TMA) construction of BBD samples

After centralized review of H&E stained slides, we retrieved archived FFPE benign breast biopsy blocks for participants. H&E sections of the corresponding FFPE tissue blocks were re-reviewed by a single pathologist to identify areas of benign proliferative lesions and normal terminal duct-lobular units (TDLUs), and to identify the areas from which the cores for the TMAs would be taken. Normal TDLUs were regions of histologically normal tissue that may or may not be adjacent to benign lesions (e.g., atypical ductal hyperplasia, usual ductal hyperplasia) [12]. TMAs were constructed at the Dana Farber/Harvard Cancer Center (DF/HCC) Tissue Microarray Core Facility by obtaining 0.6-mm cores from benign lesions and TDLUs. For each woman, up to 3 cores of normal TDLU were included in the TMA blocks. We previously evaluated our TMA construction methods and confirmed a high success rate (76%) of capturing normal TDLUs in these TMA blocks [32].

### Immunohistochemistry (IHC) for stem cell markers

The expression of the stem cell markers was evaluated by semi-automated IHC technique that allows the quantification of markers’ expression levels and localization of the target signal to specific cells/structures. For each of the three markers one 5-μm paraffin section was cut from a single TMA block and then stained at the University of Florida Pathology Core Lab on DAKO AutostainerPlus according to the previously standardized protocol with commercial antibodies (CD44 [DAKO] 1:25 dilution; CD24 [Invitrogen] 1:200 dilution and ALDH1A1 [Abcam] 1:300 dilution) [33–35]. Briefly, slides were de-paraffinized with xylene and re-hydrated through decreasing concentrations of ethanol to water, including an intermediate step to quench endogenous peroxidase activity (3% hydrogen peroxide in methanol) and transferred to 1X TBS-T (Tris-buffered saline-Tween). For heat-induced antigen retrieval, sections were heated in a steamer while submerged in Citra (Biogenex, Fremont, CA) or Trilogy (Cell Marque, Rocklin, CA) for 30 min. Next, slides were (1) rinsed in 1XTBS-T and incubated with a universal protein blocker Sniper (Biocare Medical, Walnut Creek, CA) for 10 (for CD44 and ALDH1A1) or 15 min (for CD24); (2) rinsed in 1XTBS-T and co-incubated in primary antibody ALDH1A1, CD24, or CD44 for 1 h; and (3) rinsed in 1XTBS-T followed by application of conjugated secondary antibody (Mach 2 goat anti-rabbit horse [or mouse] radish peroxidase-conjugated, Biocare Medical, Walnut Creek, CA) for 30 min. Detection of antibodies was achieved by incubating slides in 3′3′ diaminobenzidine (Vector Laboratories Inc., Burlingame, CA) for 4 min. Slides were counterstained with hematoxylin (Biocare Medical, Walnut Creek, CA) 1:10 for 3 min and mounted with Cytoseal XYL (Richard-Allen Scientific, Kalamazoo, MI). The laboratory implemented standard quality control procedures.

### Image analysis

Stained TMA sections were digitized at 40× using the PhenoImager HT (Akoya Biosciences, Marlborough, MA). QuPath v0.5.0 was used to quantify the immunoreactivity of the markers [36]. For each marker, we randomly selected 12 tissue cores of variable staining intensities on a TMA to train tissue segmentation into epithelium and stroma, and determine the minimum intensity to score a cell as immunoreactive [37]. We used random forest object classifier and the “positive cell detection” command to optimize cell parameters, intensity thresholds for hematoxylin and cytoplasm (mean optical density), and used the default values for all other parameters.

We focused the current analysis on the expression of stem cell markers in normal TDLU cores for the following reasons: (1) we specifically targeted normal TDLUs in construction of these TMAs and thus the number of women with benign lesion cores was smaller and would not allow to draw meaningful conclusions; (2) in our earlier reliability study, we observed higher heterogeneity within benign lesion cores as they were represented by various lesion types [33]; and (3) we were interested in the underlying changes in the breast tissue happening early in the process of breast carcinogenesis and thus normal TDLUs were more relevant to address our research questions.

### IGF-1 and IGFBP-3 laboratory assays

IGF-1 and IGFBP-3 levels were assayed in two batches for luteal and untimed samples in the NHSII by ELISA after acid extraction at the Department of Medicine and Oncology at McGill University, using reagents from Diagnostic Systems Laboratory (Webster, TX, USA). The mean coefficients of variation were 3.5 and 2.8% for IGF-1 and 1.6 and 3.7% for IGFBP-3 [28].

### Covariate information

Information on breast cancer risk factors was obtained from the biennial questionnaires closest to the biopsy date. Women were considered to be postmenopausal if they reported: (1) no menstrual periods within the 12 months before biopsy with natural menopause, (2) bilateral oophorectomy, or (3) hysterectomy with one or both ovaries retained, and were 54 years or older for ever smokers or 56 years or older for never smokers [38, 39].

### Statistical analysis

To account for batch-to-batch variation, IGF-1 and IGFBP-3 levels were recalibrated to have a comparable distribution to an average batch according to methods outlined by Rosner et al. [40]. We used generalized linear regression to examine the associations of plasma concentrations of IGF-I and IGFBP-3 (continuous log-transformed and batch-specific quartiles) with log-transformed expression of each marker (in epithelium and stroma, measured as weighted [by the number of cells] average across available cores for a woman), adjusted for the following covariates: (1) Model 1: age (continuous), BMI (continuous); (2) Model 2 (full): age (continuous), BMI (continuous), a family history of breast cancer (yes/no), menopausal status (premenopausal, postmenopausal, unknown menopausal status), age at menarche (< 12, 12, 13, > 13, unknown), combined parity/age at first birth (parous with first birth before age 25, parous with first birth at or after age 25, nulliparous, unknown), benign breast disease subtype (non-proliferative, proliferative without atypia, proliferative with atypia), alcohol use (none, > 0 − < 5, ≥ 5 g/day), and NHS cohort (NHS, NHSII); and (3) Model 3 (reduced): age (continuous), BMI (continuous), menopausal status (premenopausal, postmenopausal, unknown menopausal status), combined parity/age at first birth (parous with first birth before age 25, parous with first birth at or after age 25, nulliparous, unknown), and benign breast disease subtype (non-proliferative, proliferative without atypia, proliferative with atypia). Due to the relatively small sample size and large number of covariates, we present Model 3 as our final model. Median levels within respective categories of IGF-1 and IGFBP-3 were used for the test of trend. All the analyses were performed using SAS software (version 9.4, SAS Institute, Cary, NC). All tests of statistical significance were 2-sided.

## Results

In this study of 151 women, 3 (2.0%) had non-proliferative disease, 137 (90.7%) had proliferative disease without atypia, and 11 (7.28%) had atypical hyperplasia. The average age at biopsy was 42.9 years (range 34–61). Majority of the women were premenopausal (88.7%). Age-adjusted characteristics of women in the study by IGF-1 and IGFBP-3 levels are presented in Table 1.

**Table 1:** Age-adjusted characteristics of Nurses’ Health Study II participants, by IGF-1 levels at time of biopsy

Characteristic	Below median IGF-1 n = 76	Above median IGF-1 n = 75	Below median IGFBP-3 n = 75	Above median IGFBP-3 n = 76
Mean (SD)				
Age (years)^a^	44.62 (5.09)	41.25 (4.59)	43.92 (5.44)	41.99 (4.61)
Age at menarche (years)	12.51 (1.43)	12.79 (1.30)	12.72 (1.56)	12.76 (1.42)
Body Mass Index (kg/m^2^)	26.51 (6.65)	24.47 (4.00)	25.22 (5.32)	25.55 (5.48)
Alcohol, g/day	2.67 (4.32)	2.92 (5.06)	3.28 (5.84)	2.42 (3.74)
Percentages				
Parity/age at first birth				
Nulliparous	20	15	22	14
Parous, age < 25 years	31	27	27	28
Parous, age ≥ 25 years	47	56	49	57
Family history of breast cancer	5	5	4	9
Benign breast disease				
Non-proliferative	4	0	3	1
Proliferative without atypia	87	94	87	95
Proliferative with atypia	9	6	10	4
Menopausal status/hormone use				
Premenopausal	84	93	86	94
Postmenopausal	14	3	11	2

^a^Value is not age adjusted

Age and BMI-adjusted risk estimates (Model 1) are presented in Supplementary Table 1 and the results of the fully-adjusted model (Model 2) are presented in Supplementary Table 2. In multivariate analysis (Table 2), circulating IGF-1 was not associated with the expression of any of the markers in epithelium or stroma (Table 2). Continuous measures of IGFBP-3 were not associated with the expression of CD44 or ALDH1A1 markers in epithelium or stroma. However, there was a suggestion of an inverse association between IGFBP-3 quartiles with stromal CD24 expression in normal TDLUs (β for 4th vs. 1st quartile = − 0.38, 95% CI − 0.69, − 0.08, *p*-trend = 0.06).

**Table 2:** Associations of IGF-1 and IGFBP-3 with log-transformed expression of stem cell markers in benign breast biopsy samples (β coefficients and 95% Confidence intervals)

IGF/IGFBP-3	CD44				CD24				ALDH1A1			
	N	In Epithelium	N	In Stroma	N	In Epithelium	N	In Stroma	N	In Epithelium	N	In Stroma
IGF-1, continuous log-transformed	147	0.09 (− 0.45; 0.62)	148	0.88 (− 0.30; 2.06)	141	0.03 (− 0.16; 0.21)	143	0.22 (− 0.20; 0.65)	145	0.28 (− 0.49; 1.06)	143	0.36 (− 1.12; 1.85)
IGF-1 quartiles												
Quartile 1	38	Ref	38	Ref	36	Ref	37	Ref	37	Ref	36	Ref
Quartile 2	35	− 0.06 (− 0.46; 0.34)	36	0.69 (− 0.17; 1.55)	33	0.02 (− 0.12; 0.16)	34	0.13 (− 0.18; 0.44)	36	0.35 (− 0.23; 0.93)	36	0.33 (− 0.79; 1.46)
Quartile 3	37	− 0.08(− 0.48; 0.32)	37	0.28 (− 0.58; 1.14)	37	− 0.02 (− 0.15; 0.12)	37	− 0.01 (− 0.32; 0.30)	36	0.20 (− 0.38; 0.78)	35	0.59 (− 0.54; 1.72)
Quartile 4	37	− 0.03 (− 0.48; 0.41)	37	0.93 (− 0.03; 1.89)	35	0.01 (− 0.14; 0.17)	35	0.25 (− 0.10; 0.60)	36	0.56 (− 0.08; 1.20)	36	0.54 (− 0.72; 1.79)
p-trend^a^		0.59		0.36		0.66		0.57		0.45		0.25
IGFBP-3 continuous log-transformed	147	− 0.04 (− 0.94; 0.85)	148	− 0.41 (− 2.35; 1.53)	141	− 0.17 (− 0.47; 0.13)	143	− 0.60 (− 1.30; 0.10)	145	0.25 (− 1.02; 1.52)	143	0.96 (− 1.46; 3.38)
IGFBP-3 quartiles												
Quartile 1	35	Ref	36	Ref	34	Ref	36	Ref	36	Ref	36	Ref
Quartile 2	37	− 0.11 (− 0.47; 0.26)	37	− 0.15 (− 0.93; 0.62)	35	− 0.09 (− 0.21; 0.04)	35	− 0.27 (− 0.55; 0.02)	36	− 0.19 (− 0.71; 0.33)	35	0.28 (− 0.73; 1.28)
Quartile 3	37	0.06 (− 0.32; 0.44)	37	0.10 (− 0.72; 0.91)	34	− 0.06 (− 1.19; 0.08)	34	− 0.22 (− 0.52; 0.07)	35	0.05 (− 0.49; 0.59)	35	− 0.23 (− 1.27; 0.81)
Quartile 4	38	− 0.12 (− 0.51; 0.27)	38	− 0.58 (− 1.42; 0.26)	38	− 0.08 (− 0.21; 0.05)	38	− 0.38 (− 0.69; − 0.08)	38	− 0.18 (− 0.73; 0.38)	37	0.11 (− 0.98; 1.20)
p-trend^a^		0.82		0.52		0.56		0.06		0.21		0.77

Adjusted for age (continuous), BMI (continuous), parity/age at first birth (nulliparous, parous women with age at first birth < 25, parous women with age at first birth ≥ 25), menopausal status (premenopausal, postmenopausal, unknown), and benign breast disease subtype (non-proliferative, proliferative without atypia, proliferative with atypia)

^a^*p*-trend test performed using median values within each quartile

## Discussion

In this study of 151 women, we only found significant inverse associations between circulating IGFBP-3 with stromal CD24 expression in benign breast tissues. IGF-1 is expressed in many tissues and in the breast, predominantly in the stroma of normal and malignant tissue [41]. IGF-1 signaling pathway plays a critical role in both the EMT and stem cell-related processes in normal and cancerous tissues with numerous studies showing its involvement in self renewal, stem cell surface markers, migration, and invasion and tumor initiation in lung, prostate, liver, and breast cancers [13]. Previous pooled analyses reported positive associations of circulating IGF-1 and IGFBP-3 with breast cancer [42]. Stem cell markers CD44, CD24, and ALDH1A1 in breast tumors were linked to younger age at diagnosis, unfavorable tumor characteristics, (i.e. grade, stage, and triple-negative status), metastatic spread, poor prognosis and chemotherapy resistance [16–24], with positive associations for CD44 and ALDH1A1 and inverse associations for CD24. Our current study observed a suggestive inverse association between circulating IGFBP-3 and benign breast stromal CD24 expression, providing indirect evidence of the effect of IGF on EMT and stem cell activity in normal TDLUs.

To our knowledge, this is the first study to date exploring the associations of IGF-1 and IGFBP-3 with breast stem cell markers. The analysis used data from NHSII, an established cohort with more than 30 years of follow-up, confirmed benign breast disease status, and comprehensive information on breast cancer risk factors. Our study has a few limitations. First, we had only a single IGF-1 and IGFBP-3 measurement. However, previous work in this cohort shows high correlations of these measures over the 3-year period among premenopausal women (interclass correlations 0.70 for IGF-1 and 0.74 for IGFBP-3), suggesting that a single measurement of IGF-1 and IGFBP-3 reliably represents their levels over a long period of time [28]. Next, we recognize that biopsy samples come from a specific area of the breast. Our previous work shows that this sampling method is able to provide strong evidence for a priori hypotheses and meaningful findings for breast tissue involution [5], identification of markers associated with breast cancer risk [12, 43, 44], and associations with known breast cancer risk factors, suggesting that this limitation has minimal impact on research findings [45]. Further, previous studies suggest that CD44( +)CD24(-/low) and ALDH1(high) expression could be used to characterize two largely non-overlapping populations of breast cancer stem cells which have epithelial-like and mesenchymal-like phenotypes, respectively [46–48]. We stained each marker separately. Hence, we were unable to determine the co-localization of these stem cell markers or assess the combination of these markers’ expression on a cell-by-cell basis. Finally, we were unable to make any adjustments for circulating estrogens to account for interplay between estrogens and IGF pathway and their potential influence on stem cells as these measurements were not available in this study sample.

## Conclusions

We found a suggestive association of higher circulating IGFBP-3 levels with lower stromal CD24 expression in benign breast tissue. As stem cells are characterized by higher CD44/lower CD24 expression, our findings support the hypothesis that the activation of the IGF pathway induces stem cell activity in the breast. Future studies are needed to confirm our findings.

## Supplementary Information

Additional file 1.

## Data Availability

The data that support the findings of this study are available from the Nurses’ Health Studies, however they are not publicly available. Investigators interested in using the data can request access, and feasibility will be discussed at an investigators’ meeting. Limits are not placed on scientific questions or methods, and there is no requirement for co-authorship. Additional data sharing information and policy details can be accessed at http://www.nurseshealthstudy.org/researchers.

## Abbreviations

BBD: Benign breast disease
BMI: Body mass index
CI: Confidence interval
EMT: Epithelial-to-mesenchymal transition
IGF: Insulin-like growth factor
IGFBP-3: Insulin-like growth factor-binding protein-3
NHS: Nurses’ Health Study
TDLU: Terminal duct-lobular units
TMA: Tissue microarray
