# Supplementary material for: Associations of circulating insulin-like growth factor-1 and insulin-like growth factor binding protein-3 with the expression of stem cell markers in benign breast tissue
Source: Breast Cancer Res. 2025 Apr 7;27:53. doi: 10.1186/s13058-025-02002-z (PMC11978140; doi:10.1186/s13058-025-02002-z)
Supplement: Supplementary file 1 — Additional file 1. [file 13058_2025_2002_MOESM1_ESM.docx]

**Supplementary table 1. Age and BMI-adjusted associations of IGF-1 and IGFBP-3 with log-transformed expression of stem cell markers in benign breast biopsy samples (β coefficients and 95% Confidence intervals)**

|  | **CD44** | | | | **CD24** | | | | **ALDH1A1** | | | |
| --- | --- | --- | --- | --- | --- | --- | --- | --- | --- | --- | --- | --- |
| **IGF/IGFBP-3** | **N** | **In Epithelium** | **N** | **In Stroma** | **N** | **In Epithelium** | **N** | **In Stroma** | **N** | **In Epithelium** | **N** | **In Stroma** |
| IGF-1, continuous log-transformed | 150 | -0.03 (-0.53; 0.47) | 151 | 0.56 (-0.52; 1.65) | 144 | 0.01 (-0.16; 0.19) | 146 | 0.11 (-0.29; 0.51) | 148 | 0.18 (-0.53; 0.90) | 146 | 0.30 (-1.06; 1.66) |
| IGF-1 quartiles  Quartile 1  Quartile 2  Quartile 3  Quartile 4  p-trend ^a^ | 39  36  38  37 | Ref  -0.01 (-0.38; 0.35)  -0.06 (-0.43; 0.31)  -0.05 (-0.47; 0.37)  0.77 | 39  37  38  37 | Ref  0.57 (-0.20; 1.35)  0.18 (-0.63; 0.98)  0.76 (-0.15; 1.67)  0.48 | 37  34  38  35 | Ref  0.03 (-0.10; 0.15)  -0.01 (-0.14; 0.12)  0.03 (-0.12; 0.17)  0.80 | 38  35  38  35 | Ref  0.13 (-0.16; 0.42)  -0.03 (-0.32; 0.27)  0.20 (-0.13; 0.54)  0.75 | 38  37  37  36 | Ref  0.09 (-0.42; 0.61)  -0.03 (-0.57; 0.50)  0.43 (-0.18; 1.03)  0.51 | 37  37  36  36 | Ref  0.32 (-0.67; 1.32)  0.53 (-0.52; 1.57)  0.60 (-0.58; 1.77)  0.30 |
| IGFBP-3 continuous log-transformed | 150 | 0.01 (-0.86; 0.88) | 151 | -0.14 (-2.02; 1.74) | 144 | -0.11 (-0.41; 0.19) | 146 | -0.52 (-1.20; 0.17) | 148 | 0.19 (-1.05; 1.43) | 146 | 1.29 (-1.06; 3.64) |
| IGFBP-3 quartiles  Quartile 1  Quartile 2  Quartile 3  Quartile 4  p-trend ^a^ | 37  37  37  39 | Ref  -0.13 (-0.48; 0.21)  0.05 (-0.33; 0.42)  -0.12 (-0.50; 0.26)  0.90 | 38  37  37  39 | Ref  -0.25 (-0.98; 0.49)  0.06 (-0.73; 0.85)  -0.51 (-1.32; 0.31)  0.69 | 36  35  34  39 | Ref  -0.03 (-0.15; 0.09)  -0.02 (-0.15; 0.11)  -0.06 (-0.19; 0.07)  0.59 | 38  35  34  39 | Ref  -0.24 (-0.51; 0.03)  -0.21 (-0.50; 0.08)  -0.35 (-0.65; -0.05)  0.06 | 38  36  35  39 | Ref  -0.10 (-0.59; 0.40)  0.09 (-0.44; 0.62)  -0.18 (-0.72; 0.36)  0.30 | 38  35  35  38 | Ref  0.24 (-0.71; 1.19)  -0.18 (-1.19; 0.84)  0.21 (-0.85; 1.27)  0.48 |

^a^ p-trend test performed using median values within each quartile

**Supplementary table 2. Associations of IGF-1 and IGFBP-3 with log-transformed expression of stem cell markers in benign breast biopsy samples (β coefficients and 95% Confidence intervals) ^a^**

|  | **CD44** | | | | **CD24** | | | | **ALDH1A1** | | | |
| --- | --- | --- | --- | --- | --- | --- | --- | --- | --- | --- | --- | --- |
| **IGF/IGFBP-3** | **N** | **In Epithelium** | **N** | **In Stroma** | **N** | **In Epithelium** | **N** | **In Stroma** | **N** | **In Epithelium** | **N** | **In Stroma** |
| IGF-1, continuous log-transformed | 147 | -0.02 (-0.55; 0.51) | 148 | 0.78 (-0.39; 1.96) | 141 | -0.4x10^-2^ (-0.19; 0.18) | 143 | 0.16 (-0.27; 0.59) | 145 | 0.21 (-0.58; 0.99) | 143 | 0.39 (-1.11; 1.89) |
| IGF-1 quartiles  Quartile 1  Quartile 2  Quartile 3  Quartile 4  p-trend ^b^ | 38  35  37  37 | Ref  -0.03 (-0.43; 0.36)  -0.15 (-0.54; 0.24)  -0.11 (-0.54; 0.32)  0.90 | 38  36  37  37 | Ref  0.88 (0.01; 1.74)  0.30 (-0.55; 1.15)  0.89 (-0.06; 1.84)  0.55 | 36  33  37  35 | Ref  0.04 (-0.10; 0.17)  -0.03 (-0.17; 0.10)  -0.2x10^-2^ (-0.15; 0.15)  0.32 | 37  34  37  35 | Ref  0.17 (-0.15; 0.49)  -0.03 (-0.34; 0.29)  0.22 (-0.13; 0.57)  0.83 | 37  36  36  36 | Ref  0.35 (-0.24; 0.94)  0.12 (-0.46; 0.70)  0.50 (-0.14; 1.14)  0.68 | 36  36  35  36 | Ref  0.38 (-0.78; 1.54)  0.56 (-0.58; 1.70)  0.53 (-0.73; 1.79)  0.32 |
| IGFBP-3 continuous log-transformed | 147 | 0.18 (-0.72, 1.07) | 148 | -0.45 (-2.42, 1.53) | 141 | -0.09 (-0.40; 0.22) | 143 | -0.52 (-1.25; 0.20) | 145 | 0.48 (-0.82; 1.79) | 143 | 1.11 (-1.39; 3.60) |
| IGFBP-3 quartiles  Quartile 1  Quartile 2  Quartile 3  Quartile 4  p-trend ^b^ | 35  37  37  38 | Ref  -0.02 (-0.38; 0.34)  0.16 (-0.22; 0.55)  0.03 (-0.37; 0.43)  0.66 | 36  37  37  38 | Ref  -0.18 (-0.97; 0.61)  0.07 (-0.77; 0.90)  -0.52 (-1.39; 0.34)  0.60 | 34  35  34  38 | Ref  -0.06 (-0.19; 0.07)  -0.03 (-0.16; 0.11)  -0.03 (-0.17; -0.10)  0.83 | 36  35  34  38 | Ref  -0.26 (-0.55; 0.04)  -0.22 (-0.53; 0.09)  -0.34 (-0.66; -0.02)  0.14 | 37  36  35  38 | Ref  -0.10 (-0.64; 0.44)  0.15 (-0.41; 0.72)  -0.03 (-0.61; 0.55)  0.11 | 36  35  35  37 | Ref  0.29 (-0.76; 1.33)  -0.15 (-1.24; 0.93)  0.19 (-0.95; 1.33)  0.70 |

^a^ Adjusted for age (continuous), BMI (continuous), age at menarche (<12, 12, 13, >13), a family history of breast cancer (Yes/No), parity/age at first birth (nulliparous, parous women with age at first birth<25, parous women with age at first birth≥25), menopausal status (premenopausal, postmenopausal, unknown), benign breast disease subtype (non-proliferative, proliferative without atypia, proliferative with atypia), and alcohol use (none, >0-<5, ≥5 g/day)

^b^ p-trend test performed using median values within each quartile
